# Immunological Disorders: Gradations and the Current Approach in Laboratory Diagnostics

**DOI:** 10.3390/pathophysiology32020017

**Published:** 2025-04-18

**Authors:** Anna A. Starshinova, Andrey An. Savchenko, Alexander Borisov, Igor Kudryavtsev, Artem Rubinstein, Irina Dovgalyuk, Anastasia Kulpina, Leonid P. Churilov, Polina Sobolevskaia, Tamara Fedotkina, Dmitry Kudlay, Evgeny V. Shlyakhto

**Affiliations:** 1 Department of Mathematics Computer Science, St. Petersburg State University, 199034 St. Petersburg, Russia; asya.starshinova@mail.ru; 2 Medicine Department, St. Petersburg State University, 199034 St. Petersburg, Russia; l.churilov@spbu.ru (L.P.C.); dr.polinasobolevskaia@bk.ru (P.S.); 3 Almazov National Medical Research Centre, 197341 St. Petersburg, Russia; igorek1981@yandex.ru (I.K.); arrubin6@mail.ru (A.R.); t.v.fedotkina@gmail.com (T.F.); e.shlyakhto@almazovcentre.ru (E.V.S.); 4 Federal Research Center «Krasnoyarsk Science Center» of the Siberian Branch of the Russian Academy of Sciences, Scientific Research Institute of Medical Problems of the North, 660036 Krasnoyarsk, Russia; aasavchenko@yandex.ru (A.A.S.); 2410454@mail.ru (A.B.); 5 Department of Immunology, Institution of Experimental Medicine, 197376 St. Petersburg, Russia; 6 Research Institute of Phthisiopulmonology, 190961 St. Petersburg, Russia; prdovgaluk@mail.ru; 7 Laboratory of Comparative Sensory Physiology, Sechenov Institute of Evolutionary Physiology and Biochemistry of the Russian Academy of Sciences, 194223 St. Petersburg, Russia; 8 Medical Department, I.M. Sechenov First Moscow State Medical University, 197022 Moscow, Russia; d624254@gmail.com; 9 Department of Pharmacology, I.M. Sechenov First Moscow State Medical University, 197022 Moscow, Russia; 10 Institute of Immunology FMBA of Russia, 115478 Moscow, Russia; 11 Department of Pharmacognosy and Industrial Pharmacy, Faculty of Fundamental Medicine, Lomonosov Moscow State University, 119991 Moscow, Russia

**Keywords:** immunopathology, immunological disorders, immune cells, laboratory diagnostics, molecules, systemic inflammatory response

## Abstract

Currently, understanding the immune response, its abnormalities, and its diagnostic possibilities is a key point in the management of patients with various diseases, from infectious to oncological ones. The aim of this review was to analyze the data presented in the current literature on immune disorders and the possibility of their laboratory diagnostics in combination with clinical manifestations. We have performed a systematic analysis of the literature presented in international databases over the last ten years. We have presented data on the possibility of diagnosing immunopathological processes due to changes in immune cells and soluble molecules involved in the pathogenesis of a wide range of diseases, as well as the determination of antibodies to detect autoimmune processes. By applying laboratory techniques such as hematology, flow cytometry, ELISA, etc., available to most clinical laboratories worldwide, clinical data on immune system dysfunction in a wide range of diseases are being collected. This process is unfortunately still very far from being completed. However, with all the diversity of accumulated knowledge, we can currently state that the pathogenesis of the vast majority of immune-mediated diseases is not yet known. At the same time, the current success in dividing immune-mediated diseases into distinct clusters based on different types of inflammatory responses that are based on the involvement of different populations of T helper cells and cytokine molecules represents significant progress. Further research in this direction seems very promising, as it allows the identification of new target cells and target molecules for both improved diagnostics and targeted therapies.

## 1. Introduction

The epidemiology of immunological disorders has been changing significantly in recent years due to the increase in autoimmune diseases, allergic reactions, immunodeficiencies, and other immune system disorders [1,2].

In recent years, there has been a significant increase in autoimmune pathologies (rheumatoid arthritis, systemic lupus erythematosus, multiple sclerosis, type 1 diabetes mellitus, etc.) Approximately 4% of the world’s population suffers from more than 80 different variants of autoimmune diseases (AD). These diseases include both systemic and tissue-specific diseases, including systemic lupus erythematosus, insulin-dependent diabetes mellitus, autoimmune hepatitis, rheumatoid arthritis, thyroiditis, Crohn’s disease, psoriasis, multiple sclerosis, and others. Autoimmunity is more common in the female than in the male population [3].

The causes of AD are attributed to genetic predisposition, environmental influences (e.g., air pollution and chemicals), lifestyle changes (diet and stress), and possibly excessive hygiene (“hygiene hypothesis”). Also, there is an increase in the number of patients with diseases associated with allergic manifestations, such as bronchial asthma, atopic dermatitis, allergic rhinitis, and food allergies. One of the reasons for the increase in this pathology is attributed to changes in the human microbiome, exposure to allergens at an early age, and changes in immune regulation [4].

The prevalence of immunological disorders varies from region to region. For example, autoimmune and allergic diseases are more common in developed countries, which may be related to lifestyle and environmental factors. At the same time, developing countries have a higher prevalence of infectious diseases, which may affect the immune status of the population [1,5].

Immunopathology deals with the diseases of the immune system per se, as well as with the abnormal states induced under the influence of factors affecting it, and with the autoimmune and allergic diseases caused by excessive or perverted immune response, altering other organs and systems [1,2]. Nowadays, the diseases of the immune system are graded in clinically similar groups, although they have different immunopathological mechanisms [3,4,5].

Diseases of the immune system, manifested by various symptoms and signs (syndromes), disrupt the normal functioning of the body by affecting the immune-mediated and neuroendocrine regulation [4]. Hence, in clinical practice, it is necessary to determine the presence and characterize certain immunopathological syndromes, assess the degree of its severity, identify the cause of its occurrence, conduct additional laboratory and instrumental studies, and form an exact immunological diagnosis. Similarly, in other branches of Internal Medicine, a physician needs to not only recognize a concrete nosological entity (let us say, ischemic heart disease or viral hepatitis B) but also evaluate the presence and degree of heart failure (or hepatic failure) as a syndrome. Therefore, after clinical examination and obtaining laboratory tests, it is necessary to classify the immune disorders.

Diagnosis of immune disorders is associated with a number of problems. This is due to the complex multilevel organization of the immune system and the high reliability of immunity with numerous compensatory mechanisms and regulatory links between the components of the immune system and the neuroendocrine apparatus. The immune response (provided it is routinely performed, properly targeted, optimally regulated, and having adequate intensity) is minimally perceptible clinically, and its beneficial effects are incomparably greater than its costs. However, too strong, poorly regulated, and/or too weak immune response serves as a mechanistic basis for immunopathological diseases.

Traditionally, all immune system dysfunctions are classified based on their manifestations [1,6,7]. In the global medical practice, it is accepted to distinguish (1) primary immunodeficiencies, (2) secondary immunodeficiencies (immunosuppressive states), (3) autoimmune diseases, (4) autoinflammatory diseases, (5) allergic diseases, and (6) neoplastic diseases of the immune system [4,5,8].

In all of these cases, practically, a physician does not diagnose the dysfunction of the immune system per se but rather recognizes, instantly, certain nosological units, which, as a rule, have the mosaic pathogenesis, including not a single but several different types of immunopathological reactions. Taking into account the clonal selection principle of the immune response and multidirectional changes in the reactivity of its various elements, quite often even hypoergic and hyperergic modes of reactions can be combined in the same nosological entity [8,9].

It should be noted that many clinical forms of immune diseases are based on primary disorders of the immune system, compensated up to a certain time at the expense of normal or high functional activity of other components of the immune–neuroendocrine apparatus. This compensatory function has its own significance in the form of secondary lesions and symptomatic effects [5,10,11].

Indeed, this is only a private immunopathological manifestation of the general pathological law of relative expediency and potential pathogenicity of any defense reaction. The pathogenicity of defense will be the more pronounced the greater the price of adaptation and the more severe the discrepancy between the applied protective–compensatory stereotypes and the specific non-stereotypical situation [11].

Historically, immunopathological disorders are often simplistically defined in medical routine as hyporesponsive and hyperresponsive states. This division was previously widely and habitually used. However, this formalization is not always appropriate, since, for example, in the classical form of antibody-mediated hypersensitivity—immune complex reaction and its corresponding immune complex-mediated lesions on various organs—the basis of pathogenesis is precisely the hyporesponsive insufficiency of the immune complex clearance function. It may occur due to defects in macrophages; complement system factors or their cell receptors, leading to incomplete elimination of immune complexes; or their deposition on the endothelium (as a rule, in those areas of the bloodstream where the vessels are tortuous and blood pressure is relatively high). Ultimately, this leads to the development of inflammation in the form of vasculitis of various localization and severity. Thus, this disorder, in substance, is not so much a hyperreaction as an insufficient hyporeaction of the immune system macrophagal–phagocytic link [12,13].

**Figure 1:**
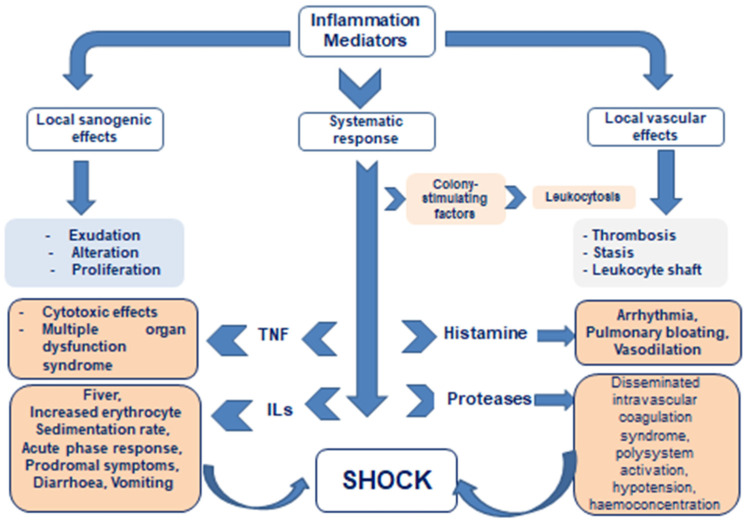
Local versus systematic action of inflammatory mediators.

A terminological problem connected with the naming of immunopathological syndromes is also of the essence because, quite often, different names for the phenomena/syndromes of the same pathogenesis are given, sometimes not consistent with the thesaurus of general pathology. Multiple names may depend on the degree of a syndrome’s severity or on the prevalence of certain symptoms, and may be given in the spirit of traditions inherent in certain national medical schools or even within the professional slang spoken by different medical specialists. For example, recently, in addition to the general concept of “systemic inflammatory response”, other close definitions and names have been coined. These are “cytokine storm” or “cytokine tempest”, “secondary hemophagocytic lymphohistiocytosis (HLH) syndrome”, “macrophage hyperactivation syndrome (MAS)”, “virus-induced hyperinflammatory syndrome”, etc. (Figure 1).

But at the heart of all differently named syndromes lies congenital and/or acquired disorders of immune response regulation and failure of barrier functions of inflammation, leading to sharply excessive systemic action of pro-inflammatory autacoids (including cytokines); abnormal activation of immune system cells, i.e., to hyperergic acute phase response, passing into *toxic–septic shock* with impaired blood circulation and respiration; and poor perfusion of organs, including those not affected by primary inflammatory foci.

The consequence is hypoxic necrobiosis of their cells with the release of additional portions of pro-inflammatory mediators and, as a result, occurrence of the multiorgan failure [14,15,16].

It means that the abovementioned names in no way belong to separate pathological processes, but only designate etiologically special variants of typical pathological processes that have long been described in general pathology: acute phase response and shock.

## 2. Local Immune Response and Its Interaction with Systemic Defense Processes

The response of the organism as a system to damage is complex and multileveled, but it remains within programmed stereotypes of its reactivity. These stereotypes include more ancient ones, inherent in the response of cells as elements of the organism, as well as newer ones, formed during the evolution of higher animals. The more ancient ones depend on bioregulators of short-range, juxta- and paracrine action produced by the cells themselves (these are known in pathophysiology as mediators of inflammation or, more broadly, in relation to both health and disease, under the name “autacoids”). One of their assembled groups is actually the cytokines [17,18].

The newer ones depend on long-range regulators systemically produced by the neuroendocrine apparatus. These include hormones and neurotransmitters. Any serious damage causes, in parallel, two kinds of programmed responses: cellular/tissue local one and systemic/integrative one. If there is no conflict between these programs, both defense pathways are kept within the bounds of moderate pathogenicity and sufficient protective efficacy [11,19].

However, the parallel effects of these bioregulators may, under certain conditions, come into conflict, jeopardizing the optimal implementation of protective programs, especially in case of extensive damage and limited compensation resources. This leads to a mutual emaciation of their protective potential and to an increase in the price of adaptation, up to the prevalence of secondary autopathogenicity of the defense itself [20,21,22].

As long as the dynamics of inflammation is not triggered in those organs and tissues, where there is no primary local damage, the organism manages to prevent the immersion of a large mass of cells in the state of hypoxic or free-radical necrobiosis. But if the systemic–local protective equilibrium is replaced by absolute prevalence of regulators of short-distance action (cytokine storm) or, on the contrary, of systemic regulatory stereotypes (centralization of blood circulation), then the cells of the organs that have fallen into hypoxia, although there were no primary damages, themselves become sources of an increasing number of inflammatory mediators. The production of inflammatory mediators is inevitable in the process of cell degeneration and death. The significant excess of these mediators in the systemic circulation disturbs blood rheological properties, makes the endothelium sticky, shifts the balance of hemostasis and anti-hemostasis to the thrombogenic side, affects the central regulation of respiration and cardiac activity, and contributes to acidosis. Finally, hemodynamic shock is formed: multiorgan hypoxic failure results from decreased venous return and their impaired perfusion [16,23].

Consequently, excessive systemic action of inflammatory mediators is an almost obligatory component of the pathogenesis of progressive hemodynamic shock. In substance, it is the increasing systemic action of inflammatory autacoids that leads through the stage of normergic acute phase response to deeper shock-like states and ultimately to shock.

But, initially, the inflammation is always a local typical pathological process, a response of vascularized tissue to damage, controlled by signals produced within its foci, and not by bioregulators that are external to them. This process is capable of creating barriers preventing the systemic spread of pathogens, and limiting the general action of bioregulators participating in focal events. The latter is known as the informational autochthony of the inflammatory focus [11]. There is a minted formulation by the founder of the battle surgery, Sir John Hunter (1728–1793), which has not been outdated over 230 years: “Inflammation can be only into the organ, but never of an organ” [18]. Preserving its local character, inflammation prevents circulatory shock, but, losing its locality due to the failure of barriers, it triggers or aggravates the pathogenesis moving the body along the abovementioned pathway through acute phase response to circulatory shock.

The classical main local signs of inflammation, described as early as in the 1st century AD by Aulus Cornelius Celsus, are swelling, pain, redness, localized heat, and organ dysfunction, which was added to this tetrad later in the 19th century by Rudolf Virchow. However, in clinical practice, it is much more important to determine the manifestation of systemic correlates of the inflammatory response, especially in severe and widespread inflammation or in inflammation of an organ hidden from direct physical examination.

The most important diagnostic step in the diagnosis of immunopathology is the identification of local (focal) signs of inflammation versus its systemic correlates.

## 3. Systemic Inflammatory Response

In 1987, an American physician, F.B. Cerra, first called hypermetabolism and multiorgan failure in sepsis a “systemic inflammatory response” [24]; a year later, this concept was detailed by the French author P.E. Laurent [25].

Since inflammation is not only infectious, and shock is not only septic, this interpretation became universal. Then, in the early 1990s, R. Bone et al. [26] introduced the term “SIRS—systemic inflammatory response syndrome”, which gained popularity and was associated with extremely excessive systemic action of inflammatory mediators in shock-like conditions and shock of any etiology, as well as in traumatic disease.

In 1992, the American Association of Thoracic Surgeons developed criteria for this syndrome [27], from which it is clear that this is a pre-shock condition or an extreme hyperergic variant of a typical pathological process well-known in pathophysiology since the 1950s, called the “acute phase response” [28]. The latter occurs after any massive impact on Toll-like receptors (TLRs) of the innate immunity, protecting the metabolic interests of the immune system against the background of stress accompanying any acute aggression. In the case of SIRS, one can state its hyperergic, excessively strong character.

The diagnosis of SIRS initially was purely clinical, but later was added with several laboratory signs. Two of its varieties can be distinguished based on the proposed criteria (Table 1).

(I) With a predominant pro-inflammatory reaction (febrile fever, leukocytosis, and/or leftward shift of the leukocyte count);

(II) With predominance of anti-inflammatory reaction (hypothermia, leucopenia, and/or lymphopenia).

The clinical manifestations of cytokine dysregulation in SIRS are summarized from various sources and listed in Table 2.

**Figure 2:**
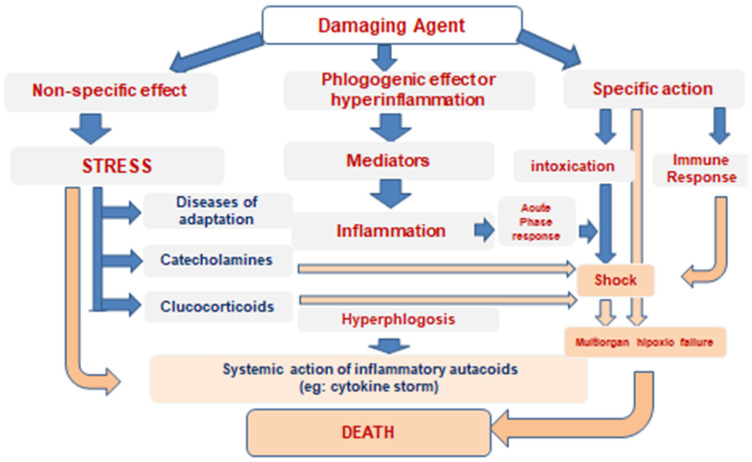
Patterns of cytokine storm development.

The two types of SIRS are often observed as phase states. This is characteristic, for example, of traumatic disease, when, in severe combined traumas against the background of shock, there is a pro-inflammatory reaction, which is replaced, due to the inertness of counterregulation, by the anti-inflammatory dominance in survivors of the acute stage of traumatic disease (Figure 2). Such phase interactions of pro-inflammatory and anti-inflammatory mechanisms in severe combined trauma and its shock consequences after the “cytokine storm” of the early phase create, in the later period, under the influence of counterregulatory mechanisms, the danger of immunosuppressive septic complications threatening the patient even after recovery from the hemodynamic shock [29]. Therefore, as it was first postulated at the end of the last century by F.A. Moore et al. [30], the multiorgan failure that develops in these states is bimodal: with an early phase dominated by SIRS and hypoperfusion hypoxia as the cause of organ failure; and a late phase, when the compensatory anti-inflammatory response syndrome (CARS) develops, driven by both anti-inflammatory factors of the immune system and the anti-inflammatory effect of glucocorticoids, derived both from the stress and iatrogenic interventions. As a result, these syndromes can be combined, and their combination is denoted as MARS (mixed antagonist response syndrome) [29]. Thus, the conflict of protective programs of different levels and orientation is clearly manifested here as well.

As a consequence of MARS in severe combined trauma, many immunosuppression manifestations developed in patients brought out of shock during the later period, despite the fact that all of them had previously undergone hyperinflammatory changes in immunoreactivity [31].

SIRS is defined when at least five diagnostic criteria (and/or mutations causing its primary development) are present. In particular, some mutations of collectin and ficolin genes predispose to the development of early SIRS in the form of neonatal sepsis [32]. The extreme degree of cytokine dysregulation is observed in the syndrome of secondary hemophagocytic lymphohistiocytosis (HLH), a term first described in rheumatology for a potentially life-threatening complication of systemic inflammatory diseases (juvenile idiopathic arthritis, systemic lupus erythematosus (SLE), Kawasaki disease, and recurrent fevers). Hemophagocytosis is defined as the engulfment by phagocytic cells of various blood cells, including erythrocytes (with ferritin concentration increase), white blood cells, and platelets [33]. In addition, the acute phase of HLH is associated with markedly elevated systemic concentrations of pro-inflammatory cytokines, which trigger a cascade of inflammatory processes and, if left untreated, lead to tissue damage and death [11,13].

Sepsis of various etiologies is directly related to cytokine storm, which serves as a key link in the pathogenesis of SIRS, as toxic–septic shock develops [34]. In this case, the triggers of the hyperinflammatory state are pathogen-associated molecular patterns (PAMPs) that stimulate pro-inflammatory events in cells of the innate immune system via Toll-like receptors. But in necrobiosis of any etiology, including those not associated with infections, degenerating cells secrete pro-inflammatory mediators and products of cellular destruction (so-called danger-associated molecular patterns—DAMPs). DAMPs also stimulate the activation and secretion of cytokines. That is why any massive cell death, and not only septic processes, is fraught with SIRS.

Noninfectious causes that can induce a systemic pro-inflammatory, particularly cytokine, response include severe trauma and burns, high-dose radiation exposure, acute pancreatitis, significant blood loss, ischemia–reperfusion syndrome, genetic disorders of the immune response (e.g., the aforementioned primary HLH), mast-cell activation syndrome in autoimmune diseases, graft-versus-host reactions, immunobiological cancer therapy with chimeric antigen receptor T-lymphocytes (CAR-T), treatment with T-lymphocyte activators, etc. [35,36,37,38].

The etiology of SIRS also includes a variety of infectious agents. The most studied infectious triggers of SIRS include “avian” influenza A(H5N1) virus, SARS-CoV-1 coronavirus associated with SARS, Middle East respiratory syndrome-associated coronavirus (MERS-CoV), SARS-CoV-2 in COVID-19, and Ebola virus [16,39].

Long-term stimulation of the immune system, even if not initially overexpressed (as in Epstein–Barr or other herpes group viruses), can also cause a cytokine storm (during the development of so-called secondary HLH—see below), as the pathogen may be a polyclonal immunostimulant and/or have superantigenic properties, inducing a cytokine response of many nonspecific lymphoid cell clones at once. Bacteria possessing superantigenic molecules, such as Staphylococcus aureus or Streptococcus pyogenes of some strains, can also induce SIRS as an element of toxic–septic shock via similar mechanism [40,41].

Regardless of the etiology, cytokine storm triggers a cascade of inflammatory processes that lead to tissue damage and secondary production of proinflammatory mediators, and, without treatment, it may be lethal [13,14,15].

Chronic excessive systemic action of inflammatory mediators not only leads to circulatory shock but is also highly pathogenic. It characterizes, first of all, the metabolic syndrome and its components, in particular, obesity and atherosclerosis, as well as systemic connective tissue dysplasia and sluggish chronic infections [19,42,43].

Identification and characterization of the SIRS, both acute and chronic, are mandatory for a physician of any speciality. Its timely recognition is a prerequisite for effective treatment.

Subsequently, without additional immunological studies, it is possible to diagnose dysfunctions of the immune system occurring in one or another link of adaptive immunity. Identification of certain immunopathological syndromes is the basis for topical diagnosis of immune disorders.

## 4. Three Main Types of Innate and Adaptive Cell-Mediated Effector Immunity

The T-cell immune response is a key component of adaptive immunity that protects the body against intracellular pathogens (viruses, and some bacteria and parasites), as well as tumor cells. T cells (T-lymphocytes) play a central role in recognizing and destroying infected or abnormal cells. Disorders of T-cell immunity can lead to immunodeficiencies, autoimmune diseases, or uncontrolled tumor growth. Today, T cells are actively used for immunotherapy, e.g., in the treatment of cancer (CAR T-cell therapy) or autoimmune diseases. The T-cell immune response provides antigen recognition. T cells recognize peptide fragments of antigens presented on MHC molecules. Cytotoxic T cells induce apoptosis with destruction of infected cells. T-helper and Treg control the strength and directionality of the immune response. After initial contact with antigen, long-lived memory T cells are formed, ensuring the formation of immune memory with the possibility of a rapid immune response upon re-infection with an infectious agent [2,3].

**Figure 3:**
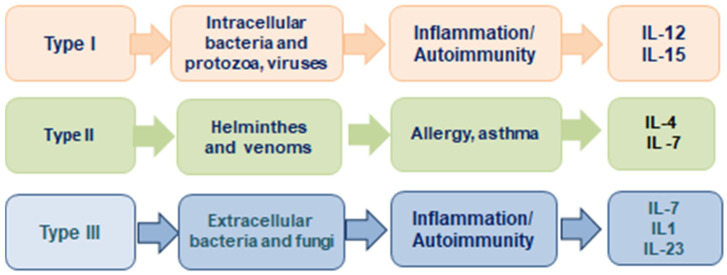
The 3 major types of innate and adaptive cell-mediated effector immunity adapted from Ref. [42].

The concept of the three cell-mediated effector functions was introduced by F. Annunziato et al. in 2015 [42]. This publication presents a new concept about effector T cells and innate lymphoid cells (ILCs) that characterize the innate and adaptive immune systems, which he proposed to classify as type 1, type 2, and type 3 (Figure 3). Type 1 immunity consists of T-bet(+) IFN-γ-producing group 1 BMPs (ILC1 and natural killer cells), CD8(+) cytotoxic T cells (TC1), and CD4(+) TH1 cells, which protect against intracellular microbes by activating mononuclear phagocytes. Type 2 immunity consists of GATA-3(+) ILC2s, TC2 cells, and TH2 cells producing IL-4, IL-5, and IL-13, which cause activation of mast cells, basophils, and eosinophils, and production of IgE antibodies, thus protecting against helminths and poisons. Type 3 immunity is mediated by retinoic acid-related orphan receptor γt(+) ILC3s; TC17 cells; and TH17 cells producing IL-17, IL-22, or both receptors, which activate mononuclear phagocytes, as well as attract neutrophils and elicit epithelial antimicrobial responses, thus protecting against extracellular bacteria and fungi. On the other hand, type 1 and type 3 immunity mediate autoimmune diseases, while type 2 reactions can cause allergic diseases.

### 4.1. Immunopathological Syndromes Associated with the First Type of Cellular Immune Response

It is known that naive T-lymphocytes, being initially capable of producing a wide range of cytokines, polarize during further differentiation and limit their signaling capabilities by a non-overlapping set of the latter. This determines their partnership with different cells and, accordingly, their different functions in immunological interactions. Th1, Th2, and Th17 subpopulations of T-helper cells migrate to the foci of inflammation, while follicular T-helper cells (fTh) remain in lymphoid organs, migrating to their follicles. According to these subpopulations of T-lymphocytes, different types of immune responses are distinguished in terms of key participants and active bioregulators, which, however, frequently intermingle in the course of immune defense [44].

### 4.2. Cellular-Effector Deficiency

This syndrome is manifested by one or more of the following signs:

- Frequent acute respiratory viral infections (more than 4 times a year);
- All types of warts, acute condylomas mediated by human papillomavirus, and molluscum contagiosum;
- Clinically evident infections caused by the Herpesviridae group (recurrent course of herpes types 1 and 2, type 3 (herpes zoster), cytomegalovirus infection, and diseases caused by Epstein–Barr virus (virus type 4) or by the viruses of viral hepatitis (B, C, D, F, and G);
- Viral enteritis;
- Recurrent childhood infections and/or infections that develop after vaccination (in children over 7 years of age and adults);
- Fungal infections (candidiasis and other mycoses of the skin, nails, and mucous membranes (thrush); trichophytosis; and visceral mycoses;
- All types of tumor processes.

It should be especially noted that any tumor development is impossible without disturbances in the cellular link of immunity. Therefore, from the standpoint of the modern concept of immunoediting, all patients with cancer, beginning from the moment of diagnosis of the disease, are considered immunocompromised individuals, having at least some disorders in the cellular immunity.

### 4.3. Cell-Mediated Damage (Delayed-Type Hypersensitivity)

Prolonged persistence of antigens within antigen-presenting cells or the presence of objects that are not eliminated by inflammation chronically stimulating the immune system (e.g., crystals) creates conditions for the development of delayed-type hypersensitivity and granuloma formation [39,44].

The main diseases with delayed-type hypersensitivity reactions include leprosy, tuberculosis, schistosomiasis, Crohn’s disease, brucellosis, syphilis, legionellosis, some mycoses (where the first and third types of immune response are combined—see below), rickettsioses, Q fever, and intracellular parasitoses (where the first type of immune response is combined with the second one—see below), a total of over 70 different infections and non-infectious diseases with persistence of the foreign bodies difficult to metabolize, and products of disturbed metabolism. Examples of non-infectious granulomatous diseases are pneumoconiosis, gout and, to a certain extent, even atherosclerosis with its deposition of cholesterol crystals and modified lipoproteins. In rheumatic fever, Aschoff–Talalaev granulomas form as noninfectious ones, although this immunopathological disease is provoked initially by certain serotypes of Streptococci (see below). Many granulomatous diseases have no conclusively proven causative agent, but they carry features of autoimmune origin, such are sarcoidosis, Crohn’s disease and nonspecific ulcerative colitis, granulomatous vasculitis, rheumatoid arthritis, De Quervain’s thyroiditis, chronic lymphocytic hypophysitis, etc. [11,45].

### 4.4. Immunopathological Syndromes Associated with the Second Type of Cellular Immune Response

Th2 cells are distinguished by the production of interleukins (IL)-4, -5, and -13 and interaction with eosinophils (via IL-5), basophils, and mast cells (with the participation of IL-4 and IL-13), involved in the driving of hyperergic exudative inflammation. Through IL-4, they promote isotypic switching of antibody formation to reagins (IgE and IgG1). Through IL-4 and IL-13, Th2 is able to polarize macrophages into non-classical anti-inflammatory M2 cells, inducers of fibrosis, and conductors of the reparative component of inflammation. The same ILs allow Th2 lymphocytes to stimulate both mucus secretion by epithelial cells and gut peristalsis. All of these effects support antiparasitic immunity, where Th2 cells play a key role, especially with regard to extracellular parasites: helminths and their larvae.

Due to the fact that the realization of this type of response belongs not only to circulating eosinophils and basophils, but also to mastocytes (tissue resident cells), the clinical manifestations of hyporesponsiveness of this type are not defined. On the contrary, a large number of diseases (allergy, if we take into account all its pathogenetic varieties, affects more than 30% of the world population) are associated with a hyperreactive (hyperergic) state, which is immediate-type hypersensitivity [46,47]. In addition, since placentation in higher mammals is an evolutionary superstructure based on modified tolerized reactions of antiparasitic immunity, Th2-dependent processes are also the most important mechanism of placenta formation and basis of the physiological pregnancy [11,44].

Th2 immune response not only plays the above-said physiological roles but also (being excessive/poorly regulated) serves as the main link in pathogenesis of many allergic inflammatory diseases (so-called atopic ones).

### 4.5. Hypersensitivity of the Immediate Type

In 1968, British immunologists R. R. A. Coombs and P. G. H. Gell proposed a classical gradation of hypersensitivity or allergic reactions, including two types (antibody-mediated (I–III) and cell-mediated (IV) and three categories of the first type—anaphylactic (I), cytotoxic (II), and immune complex allergy (III) [46].

According to this classical grading, the term “immediate hypersensitivity” includes all antibody-mediated categories of allergic reactions [48]. In view of the accumulation of a large volume of new, practically useful data and, not least, yielding to the influence of the North American school (which, unlike European ones, reduces the term “allergy” to anaphylactic category only), the European Academy of Allergy and Clinical Immunology (EAACI), in 2023–2024, agreed via consensus on a new classification of hypersensitivity reactions [49].

It fully incorporates the classical gradation by Coombs and Gell (supplementing it with categories V–VII, which classical allergology attributed to pseudoallergy or allergoid reactions, where specific lymphocytes and antibodies are not involved). According to the EAACI classification, the term “immediate hypersensitivity” covers only the reagin-dependent reactions of category I. Its development is associated with the action of antigen (allergen) on specific “homocytotropic” IgE and IgG1 located on mast cells and other cells that have a receptor for the Fc-fragment of immunoglobulins of the abovementioned classes [46].

In sensitized organisms, immediate-type reactions have a very short latent period, as contact of antigen with “charged” reactive sensor cells–reservoirs of inflammatory mediators is sufficient for their degranulation, launching the inflammation immediately. The syndrome is manifested by reactions occurring usually 5–30 min (and even less) after contact with the specific allergen (if primary immune response already gave enough homocytotropic immunoglobulins) [48,49].

It includes atopic diseases, the key link in the pathogenesis of which is enhanced IgE (IgG1)-mediated immune reactions. The classic manifestations of this type of hypersensitivity are anaphylactic reactions to foreign allergens (in the form of urticaria, Quincke’s oedema, or even systemic anaphylaxis manifested by life-threatening anaphylactic circulatory shock, especially in the case of parenteral penetration and hematogenous spread of the allergens after insect stings or drug injections). Systemic anaphylaxis requires emergency medical care. Anaphylactic forms of food allergy have also been described. In local reactions, the respiratory tract is most often affected by the formation of allergic rhinitis, sinusitis, atopic bronchial asthma (i.e., distal recurrent eosinophilic bronchitis due to bronchial hypersensitivity), and soft tissue oedema of the oral cavity and upper respiratory tract (Quincke’s oedema). Atopic kinds of conjunctivitis, dermatitis, and stomatitis are frequent. Even anaphylactic coronary spasm has been described. Drug allergy of this type is also widespread [4,7,11,50,51]. The most common triggers of drug allergy include antibiotics, non-steroidal anti-inflammatory drugs, X-ray contrast agents, and anesthetics. It should be remembered that drug allergies may also have other mechanisms related to the non-atopic categories of hypersensitivity. Moreover, the hyperreaction may be pseudo-allergic or allergoid (i.e., similar to an allergic reaction but developing without the involvement of specific antibodies/lymphocyte clones), for example, due to the direct degranulating action of drugs on mast cells and basophils. Such forms do not belong to anaphylaxis [7,11].

## 5. Immunopathological Syndromes Associated with the Third Type of Cellular Immune Response

The third type of immune response occurs with the key participation of Th17-helper cells, which require IL-1β, IL-23, TNFβ, and, above all, IL-6 for their differentiation. By signaling with their IL-17 and IL-22, these lymphocytes (sometimes subdivided into Th17+ and Th22 subgroups) interact mainly with neutrophils and epithelial cells to orchestrate inflammation directed against extracellularly localized pathogens. This group includes capsular microorganisms that do not persist in phagocytes (according to clinical and microbiological terminology, “putrefactive microflora”: Staphylococci, Streptococci, Gonococci, Meningococci, Pseudomonas aeruginosa, Proteus group, Bacillus anthracis, etc.), as well as fungi.

Another process controlled by Th17/Th22 cytokines is the growth and regeneration of epithelia, which is important in the healing of wounds, erosion, and ulcers, as well as in the control of reparative processes in solid epithelial organs (liver, kidneys, thyroid, etc.) [44,52].

Accordingly, this type of reaction includes the most effective “disinfectors” of inflammation foci and surfaces of organs and tissues, which are neutrophils with their powerful oxidative free-radical attack on the objects of phagocytosis. Only these cells have the ability to produce such a strong antimicrobial agent as hypochlorite-anion (perchlorate), and the greenish-yellow color of pus in purulent inflammation with type 3 immune response depends on the content of chromium in the active center of myeloperoxidase and tetravalent chromium salts in the exudate [11]. Clinical manifestations associated with disorders of the third type of cellular immune response are very diverse. However, from the point of view of the pathogenesis of disease development, we can distinguish the syndrome of macrophage–phagocytic link insufficiency and a group of patients with the autoinflammatory syndromes.

### 5.1. Macrophage–Phagocyte Deficiency Syndrome

The diagnostic features of this syndrome are often identical to those of the syndrome of humoral immunity deficiency (see below). However, usually in this syndrome, bacterial infections are sluggish, without fever and other signs of acute phase response. Recurrent abscesses of various localization and local bacterial infections are considered to be characteristic signs of macrophage–phagocytic link insufficiency [2,5,11]. It should be noted that the main effectors of the third type of immune response are not macrophages, but neutrophilic granulocytes, which the founder of the theory of phagocytosis, I.I. Mechnikov, called “microphages”; therefore, pathophysiologically, it would be more accurate to call this syndrome “microphage–phagocytic deficiency”. An example of the primary hereditary variant of this disorder is the so-called Job syndrome (named after the Old Testament righteous man, Job, who suffered from persistent “terrible leprosy and scabs”). In most cases, the syndrome depends on mutations in the STAT3 master gene, which controls Th17 differentiation. In addition to the abovementioned manifestations, it is characterized by a bias in the differentiation of naive T cells toward Th2, leading to an increase in type 2 immune responses, hyperproduction of IgE and manifestations of atopic diseases, in particular dermatitis. Thus, this is a typical example of a combination of hypofunction of one and associated hyperfunction of another link of immunity, illustrating the inconsistency of a one-sided interpretation of immunopathological diseases as clearly hyper- or hyporeactive.

### 5.2. Autoinflammatory Syndromes

Autoinflammatory syndromes are pathogenetically associated with abnormal activation of innate immunity, clinically manifested by recurrent episodes of fever with acute phase response and, in severe course, provoking SIRS. It may cause various types of skin and/or mucosal and/or joint lesions [13,53]. The most typical autoinflammatory syndromes are based on abnormalities in the self-assembly and regulation of the functions of inflammasomes, provisional cytokine-producing organelles that are triggered by the action of various ligands associated with infection and cell damage on Toll-like receptors of the innate immune system cells. Many of these diseases have a genetic basis. An example of a monogenic autoinflammatory hereditary disease is Blau syndrome: early granulomatous dermatitis with uveitis and arthritis due to an autosomal dominant defect in the NOD2/CARD15 gene on chromosome 16, encoding an intracellular receptor for bacterial peptidoglycans. The inflammasome mechanism is excessively active in these patients. Familial Mediterranean fever is associated with an autosomal recessive defect of another gene on chromosome 16, MEFV, encoding the protein pyrin (marenostrin), involved in the self-assembly of inflammasomes. The disease manifests itself in attacks of fever against the background of symptoms of an acute phase response with polyserositis and myalgia. At the same time, there are many polygenic autoinflammatory diseases with additive inheritance and a threshold effect on external or endogenous triggers initiating reactions of the innate immune system. The autoinflammatory mechanism is involved in their pathogenesis, although it is not exclusive, combining with other immunopathological links. These are gout and pseudogout, Crohn’s disease and nonspecific ulcerative colitis, sarcoidosis, and juvenile idiopathic arthritis and its adult form. Autoinflammatory elements (along with autoimmune and others) are seen in the pathogenesis of Behçet disease, psoriatic arthritis, ankylosing spondylitis, and even in atherogenesis [44,54,55].

Some autoinflammatory diseases (for example, ulcerative colitis) increase the risk of neoplasms; individuals predisposed to autoinflammatory diseases can have an inadequately strong and self-damaging response to infections, including COVID-19. Interestingly, in Omenn syndrome (autosomal recessive defects of the RAG-1 and RAG-2 genes), manifestations of primary combined immunodeficiency and autoimmune/autoinflammatory symptoms are combined [44].

In real clinical practice, autoinflammatory diseases are quite diverse; their diagnosis is difficult and often delayed in time. Their main manifestations are recurrent episodes of acute phase response with manifestations of moderate SIRS (fever; aseptic inflammation of serous membranes, joints, tonsils, and skin; increased ESR, with shifts in plasma proteinogram; and leukocytosis). Chronic long-term courses of autoinflammatory syndromes contribute to the pathogenesis of amyloidosis.

## 6. Immunopathological Syndromes, Associated with Disorders of the Humoral Link of the Immune Response

The division of immunity into cellular and humoral immunity is, to a certain extent, conventional and represents a tribute to the historical heritage of immunology. In fact, antibody formation depends on the functions of cells: B-lymphocytes and their descendants—plasma cells—as well as fTh-lymphocytes (follicular T-helper cells), coordinating their affinity maturation in lymphoid follicles. On the other hand, nominal carriers of “cellular immunity”, such as T lymphocytes, secrete numerous cytokines, and the latter are soluble humoral bioregulators important for immune defense. With this caveat, we can assume that the humoral link is de facto antibody formation and antibody-dependent effector functions, including those performed by the complement system and the so-called K cells.

A large group of diseases is associated with disorders of humoral immunity. If the immune response is insufficient, as a rule, there are bacterial infections (osteomyelitis, bacterial pneumonias, purulent tonsillitis, adnexitis, meningitis, and others—up to the degree of severity causing sepsis); if the immune response is hyperresponsive, there are manifestations of the antibody-dependent cytotoxic hypersensitivity.

### 6.1. Humoral-Effector Immunodeficiency (That Is, Insufficiency of Antibody Formation and/or Antibody-Dependent Complement Functions) Is Suspected If the Patient Has Certain Symptoms

- Bacterial infections of the upper respiratory tract and ENT organs (more than 3–4 times a year with a protracted course, with residual phenomena in the form of subfebrile, asthenia, or long-lasting sore throat);
- Bacterial infections of the lungs (chronic bronchitis with or without bronchospasm, pneumonia of various etiologies);
- Bacterial infections of the skin and subcutaneous tissue (furunculosis, abscesses, phlegmons, and recurrent paraproctitis);
- Infectious–inflammatory diseases of the genitourinary system (cystitis, pyelonephritis, adnexitis, etc.);
- Other bacterial infections (meningoencephalitis, arthritis, and septicemia);
- Diseases of the digestive tract caused by bacteria (stomatitis, periodontitis, gastritis, gastroduodenitis, peptic ulcer, colitis, enterocolitis, cholecystitis, and peritonitis), or various kinds of dysbacteriosis.

### 6.2. Antibody-Dependent Cytotoxic Syndrome

Cytolytic or antibody-dependent cellular cytotoxicity (ADCC) is an important mechanism of interaction between adaptive and innate immunity. Cells that have a receptor for Fc-fragments of antibodies are able to attach to an antibody-labeled object and perform a cytolytic or cell death, inducing action against such objects without phagocytosing them. The collective name of cells capable of ADCC is K cells. K cells are not a family, a dynasty, or cell line, but an “occupation”. These are natural killers (NK cells), eosinophils, neutrophils, and macrophages—all able to act in this role. ADCC is an important mechanism of immune protection against intracellular pathogens and parasitic Protozoa, is part of antitumor immunity, and also participates in transplant rejection. But with excessive activation and incorrect targeting, the process can be harmful. In autoimmune diseases (chronic active hepatitis, nonspecific ulcerative colitis, thyroiditis, etc.), when antibodies are directed against antigens of self cells, ADCC becomes a mechanism of self-damage [11]. Hence, ADCC is observed mostly in autoimmune disorders. But antibodies against alien antigens can also trigger inflammation through the mechanism of ADCC, for example, in molecular mimicry. Molecular mimicry is one of the main mechanisms by which microorganisms can induce autoimmune responses in the host. It is defined as the presence of similar epitopes between microbial antigens and host autoantigens, leading to the activation of autoreactive T and B cells in the host organism [54,55,56]. As a rule, there are mimicking sequential determinants of common peptides, and, accordingly, the activation of “anti-alien” T-helper cells and their interaction with “anti-self” B lymphocytes occur. A similar effect can also be provoked by noninfectious mimicking antigens. Thus, a peptide from the composition of cow’s milk, albumin, mimics the peptide of human insulin. Due to that fact, artificial feeding in the first months of life increases the risk of autoimmune type I diabetes mellitus [1,12]. A classic example is rheumatic fever, in which antibodies directed against streptococcal antigens cross-react with structurally similar antigens in the heart, provoking rheumatic carditis.

ADCC leads to cell lysis, tissue damage, and/or loss of organ function. These reactions in sensitized organism usually take 2 to 24 h to develop clinical manifestation.

Antibody-mediated tissue damage can result from three different mechanisms:

- ADCC of various K cells (see above);
- Complement activation;
- Opsonizing effect and antibody-dependent phagocytosis by professional phagocytes.

In pathology, in normal conditions, and in experiments, the presence of antibodies and autoantibodies blocks or stimulates cellular receptors. Thus, research has demonstrated that they are capable of stimulating or suppressing genetically determined cellular functions and influencing the growth and vital activity of target cells [12,55].

## 7. Multifactorial Immunopathological Syndromes

As a rule, several immunopathological processes participate in the pathogenesis of a particular immunopathological disease. Therefore, it is possible to attribute them to a single pattern only in a simplified way. Thus, one should proceed from their multifactorial nature.

### 7.1. Syndrome of Pathogenic Effects of Immune Complexes

Immune complexes are formed in the body all the time and with each immune response. However, if there are any disturbances in their clearance system, they settle on the endothelium of the microcirculatory bed or accumulate perivascularly, triggering inflammation. A significant proportion of individuals (at least 5%) have hereditary or acquired defects in the immune complex clearance system: abnormalities in the structure of Fc-fragments of immunoglobulins, Fc-receptors of phagocytes, CR1 complement receptor, defects or deficiency of complement factors that act as opsonizers during the removal of immune complexes, etc. Such individuals are predisposed to immunopathological reactions associated with immune complexes (category III of hypersensitivity by Gell and Coombs) [11,44].

The syndrome of pathogenic action of immune complexes thus occurs if they have not been adequately removed by cells of innate immunity and have contributed to the formation of inflammation at the sites of their deposition [4,57]. The development of this syndrome is usually associated with chronic persistent infections, autoimmune diseases, and the influx of a large number of antigens into the sensitized or intact organism (e.g., in serum sickness).

Immune complexes, if their clearance is delayed, are deposited primarily where the blood pressure is relatively high and the vessels are tortuous; the process is facilitated by turbulent blood flow. Therefore, typical forms of immune complex pathology are glomerulonephritis, uveitis, chorioiditis, retinitis, and arthritis.

The immune complex mechanism is involved in the pathogenesis of endocarditis. Encounter diffusion of inhaled antigens of mold fungi and antibodies to them until the immune complexes settle in the equivalence zone (in the space between alveoli and capillaries of the pulmonary circulation) forms interstitial inflammation and serves as the main pathogenetic link of bronchopulmonary alveolitis [7,12]. In meningococcal infection, immune-complex reactions cause its pathognomonic sign, which is a star-shaped hemorrhagic rash. Vasculitis caused by immune complexes is one of the central links in the pathogenesis of systemic lupus erythematosus and an element of almost all rheumatological diseases [7]. Vasa-vasoritis (that is, vasculitis of the vasa vasorum in large arteries) is involved in the pathogenesis of atherosclerosis and its complications and has an immune-complex component of pathogenesis, due to which atherogenesis is accelerated in all rheumatological diseases [58].

Three stages of development of the immune complex-mediated syndrome are distinguished:

(1) Formation of mobile immune complexes;

(2) Deposition of immune complexes in tissues;

(3) Development of aseptic inflammation (quite often thromboinflammation).

### 7.2. Regeneration Fibrosis

It takes place when, after the injury, there is no compensation of the tissue defect identical to the lost tissue with restoration of the structure and ability of the organ to perform a specialized function. It is clearly manifested in chronic non-healing wounds (pressure sores, diabetic foot, etc.). However, the process of fibroplasia, i.e., replacement of lost tissue with connective one, also has its faults. Therefore, organ fibrosis should also be considered a disorder of regeneration, which means it makes sense to diagnose such a syndrome in fibrosis, and even more so in liver cirrhosis. In scleroderma (systemic sclerosis), cytokines, especially TGFβ and autoantibodies produced by the patient’s immune system, cause a sharp increase in fibrotic processes in the dermis. In systemic IgG4-associated immunopathy, a sluggish fibrosis inflammation of many epithelial organs and tissues develops against the background of an excess of antibodies of this subclass (see below). Powerful regulators of growth and regeneration addressed to both epithelial and connective tissue cells are provided by the immune system. IL-22, a product of Th17 cells and γδ T cells of the mucous membranes and innate immune cells (ILC3 and NK cells), as well as a cytokine of the IL-10 family, acts on epithelial cells (gastrointestinal tract, liver, bronchopulmonary apparatus, epidermis, and stratified squamous epithelium of the oral cavity and genitals) as a powerful mitogen, promoting their regeneration. It is produced in response to stimulation of orphan receptors of the retinoic acid family and the aryl-hydrocarbon receptor. Its synthesis is controlled by IL-23 secreted by dendritic cells and is stimulated by IL-6. On the contrary, TGFβ reduces its production. As mentioned above, erosion, aphthae, and ulcers of the mucous membranes, and non-healing skin defects are all formed with the mechanistic role of this reparation immune-dependent mechanism [52]. As for fibroplasia, cytokines stimulating fibroblast proliferation and synthesis of extracellular matrix components are supplied by M2 macrophages, controlled by Th2. Platelets also play a certain role in these processes as sources of endothelial, smooth muscle, and fibroblast growth factors, as well as epidermal growth factor, all promoting neoangiogenesis and wound healing. Moreover, there are both mitogenic and antimitogenic autoantibodies against various cells addressed to membrane or nuclear receptors that control cell growth [55], also complementing the pathogenesis of immune-dependent hyporegenerative syndrome. A striking manifestation of this syndrome can be, for example, autoimmune atrophic gastritis due to autoantibodies to the gastrin receptor and/or intrinsic antianemic factor [11].

### 7.3. Syndrome of Immune Regulatory Deficiency

It is diagnosed when the abovementioned syndromes are combined. At the same time, it is necessary to highlight some features of the diseases accompanied by this syndrome: their resistance to standard specific therapy; prolonged or chronic course with frequent and rapid relapses after treatment; activation of opportunistic microflora; mixed infections; and change in pathogen in the dynamics of the disease.

### 7.4. Autoimmune Neurological Disorders

Chronic neuropathic pain of various genesis during life is observed in 7–10% of the world population and represents a complex medical and social problem due to the limitation of working capacity and reduction in the quality of life of patients [53,54]. One of the causes of this pain syndrome is small fiber neuropathy (SFN), in which the most delicate nerve trunks—low-myelinated A delta and unmyelinated C fibers—are affected [55,56]. The incidence of NMV remains a poorly studied issue, primarily due to the difficulty of diagnosis and large-scale epidemiological studies. At present, the authors are aware of only one study that examined this issue [54,57]. The incidence of NMV of a different genesis has been shown to be 12 new cases per 100,000 people per year, with an incidence rate of about 53 patients per 100,000 people, but this issue requires further investigation with full-scale international studies [56].

NMV is often described in autoimmune and immune-mediated diseases, especially systemic lupus erythematosus, Sjögren’s syndrome, and fibromyalgia [58,59]. Given the evidence for the immune-mediated nature of sarcoidosis [9], patients with this disease can be expected to develop systemic complications, including small nerve fiber damage, most likely of cytokine-mediated genesis. According to some data, 40% to 60% of patients with sarcoidosis have some symptoms characteristic of NMV [8]. There are sporadic descriptions of cases of NMV development in patients with various infectious diseases such as HIV infection, hepatitis C, Lam’s disease, and leprosy [60,61,62].

## 8. Laboratory Diagnosis of Immune Disorders

The first stage of laboratory diagnosis is the quantitative assessment of peripheral blood cells and their morphological elements—counting the leukocyte formulas. In this analysis, attention should be paid not only to the relative but also to the absolute number of blood cells [63].

### 8.1. Assessment of Cellular Immunity Indicators

The number of leukocytes or white blood cells (WBCs) and other blood cells depends on the rate at which cells leave the bone marrow and migrate into the tissues. The processes of their margination under the influence of proinflammatory mediators and demargination under the influence of adrenal hormones also have an impact. Finally, the WBC death rate, both related to their functions or caused by autoantibodies, can influence the balance. The number of leukocytes in peripheral blood above 8 × 10^9^ cells/L is defined as leukocytosis; and below 4 × 10^9^ cells/L, as leukopenia.

Leukocytosis can be physiological because of WBC redistribution (in all kinds of stress, which stimulates disconnection of granulocytes from endothelial surface). Physiological leukocytosis is always neutrophilic and never gives left shift of WBC nuclear formula.

The main mechanism of pathological leukocytosis is a productive one due to intensified leukopoiesis. It may be neoplastic, i.e., resulting from monoclonal neoplastic proliferation (in leukemias and lymphomas), or reactive (i.e., resulted from polyclonal WBC proliferation in response to some cytokine-mediated stimulation of leukopoiesis). Polyclonal reactive leukocytosis accompanies various infections, inflammatory diseases, and some non-leukemic malignant neoplasms. The aseptic inflammation is also able to render cytokine-mediated stimulation of WBC production; hence, leukocytosis is not always a witness for infection. Allergic reactions; autoimmune diseases; burns, frostbite, and injuries; the disintegration of malignant tumors and the development of myocardial necrosis during infarction; acute onset of radiation sickness; and debut phase of intoxications killing the bone marrow cells, like in uremia, may all cause reactive leukocytosis. Severe dehydration of the organism (like in diabetic coma) causes pathologically false leukocytosis without stimulation of leukopoiesis, due to blood thickening [63].

*Leukopenia* can be either physiological (leukopenia innocens—constitutional harmless leukopenia, observed in 2–10% of practically healthy Caucasians and in major part of healthy Negroids) or pathological. Pathological leukopenia can be redistributive, which occurs from rapid margination of leukocytes due to hyperexpression of cell-adhesion molecules. An example is leukopenia in bacteremic phases of infections with Gram-negative bacteria, for example, salmonellosis. Lymphopenia, during stress, is partly associated with the homing of lymphocytes from the blood to tissues, partly with their apoptosis influenced by glucocorticoids [2,3].

True pathological leukopenia is explained by an absolute decrease in the number of leukocytes in the circulating blood, due to a significant and prolonged predominance of the rate of leukocyte death over the rate of their release into the blood. It is observed in primary aplasia and hypoplasia of red bone marrow; in secondary bone marrow insufficiency (due to irradiation, B12 and folic acid deficiencies, aplastic states of hematopoiesis, myelofibrosis, plasmacytomas, and metastases of neoplasms to the bone marrow); in hypersplenism, autoimmune diseases with antibodies to WBC, leukopenic forms, and phases of acute leukemias, due to severe infections (prolonged purulent septic ones and HIV); and in toxic aleukia caused by fungal lysosomal toxins. Leukopenia also may be iatrogenic—under the influence of drugs (e.g., amidopyrine and cytostatics) [63].

Various abnormalities of the leukocyte formula are known, which may indicate different pathological conditions in conjunction with other laboratory data.

*Neutrophilic leukocytosis* with a left shift of granulocytes’ nuclear formula can be observed because of acceleration of the leukopoiesis in bacterial infections (both nonspecific and specific), intoxication (carbon monoxide, fungi poisoning, etc.), in comas (uremic, diabetic, hepatic, etc.), after acute blood loss, and during hemolytic crisis. Leukocytosis with a shift to the right may be observed due to a decrease in or the exhaustion of leukopoiesis and aging of circulating granulocyte population: in viral and chronic bacterial infections, B12 and folic acid deficiency, bone marrow insufficiency, radiation sickness, and sepsis.

Neutropenia is usually combined with leukopenia in severe and viral infections, autoimmune and drug-induced hemocytopenias, megaloblastic and aplastic anemias, severe hypoxia, starvation, and mold toxins poisoning.

*Eosinophilia* is detected in allergic diseases; worm infestation; and skin, autoimmune, and infectious diseases during the development of clinical pictures and at the stage of recovery in malignant neoplasms and in lymphogranulomatosis, as well as in some myelogenous leukemias, Loeffler syndrome, eosinophilic granulomatosis with polyangiitis, eosinophilic fasciitis, adrenal insufficiency, and some immune complex diseases.

Eosinopenia occurs in stressful situations and hypercortisolism, acute infections, intoxications, shock, and myocardial infarction. It occurs in acute lymphoblastic leukemia, and it may occur in the post-attack period in atopic bronchial asthma.

*Basophilia* occurs in anaphylactic allergic reactions, autoimmune diseases (nonspecific ulcerative colitis, rheumatic diseases, autoimmune thrombocytopenic purpura, and immunopathological glomerulonephritis), and some helminthiases (ancylostomiasis). Interestingly, it accompanies a number of autoimmune endocrinopathies (thyroiditis and type 1 diabetes mellitus). Basophilia is characteristic of myeloproliferative diseases: true polycythemia, essential thrombocythemia, myeloid metaplasia, and chronic myelogenous leukemia. In lymphogranulomatosis, it is combined with eosinophilia. Idiopathic systemic mastocytosis can also proceed with basophilia [59]. It can be iatrogenic: with heparin treatment or in serum sickness.

Absolute lymphocytosis is observed in children up to 4 years of age as a physiological condition (and persists longer in kids with so-called lympho-hypoplastic somatotype). In adults, an increase in the number of lymphocytes is observed in infectious diseases of viral etiology (especially in infections with herpes viruses and enteroviruses), as well as in non-viral infections with persistence of pathogens within the cells (listeriosis, toxoplasmosis, whooping cough, tuberculosis, syphilis, brucellosis, mycoplasmosis, and chlamydiasis); in protozoonoses, rickettsiosis, leptospirosis, and yersiniosis; in some mycoses; and in cat- scratch disease. Non-infectious causes of lymphocytosis can be systemic and organ-specific autoimmune diseases, the “graft versus host” reaction, serum sickness, and chronic leukemia.

*Lymphopenia* is found in general and protein starvation; in some infections, particularly in the debut of typhoid fever and COVID-19, in radiation sickness, and in HIV infection with the development of acquired immunodeficiency syndrome, primary T-cell and mixed immunodeficiencies, bone marrow failure, and chronic aleukemic myelogenous leukemia; in certain stages of lymphogranulomatosis; in extremely pronounced neutrophilic leukocytoses; and in hypercortisolism and for iatrogenic reasons (use of immunosuppressants). In severe infections with lymphotropic viruses, initial lymphocytosis may be replaced by lymphopenia [2,3,63].

*Monocytopenia* is found transiently only in extremely serious leukemic and profound myeloid shifts in the white blood picture, in very severe forms of infectious diseases, being one of the signs of impaired regeneration of cells of the macrophagal lineage.

In contrast, monocytosis is detected frequently and for many reasons. In fact, the phagocytosis repertoire of macrophages is much broader than that of granulocytes. A number of pathogens are phagocytized predominantly by macrophages: mycobacteria, brucellae, salmonellae, actinomycetes, Klebsiella rhinoscleroma, Pseudomonas mallei et pseudomallei, Francisella tularensis, listeria, toxoplasmas, some fungi, and virus-infected cells of their own. Above all, macrophage phagocytosis and, consequently, monocytosis in the blood are characteristic of the response to facultative and obligate intracellular parasites [10,12,64].

The type of immune system response is calculated from the ratio of the absolute number of granulocytes and lymphocytes, and characterizes the state of innate and adaptive immunity.

In addition to the leucocyte count, it is important to determine the hemoglobin concentration, platelet and erythrocyte counts, and hematocrit and erythrocyte indices (MCV, RDW, MCH, and MCHC).

Qualitative assessment of cellular composition is possible with laser flow cytometry. Flow cytometry can be used to determine cell size, nucleus-to-cytoplasm ratio, degree of asymmetry, and fluorescence intensity [21,22,65,66]. The field of application of flow cytometry is more diverse. In addition to morphological characteristics of cells, monoclonal antibodies can be used to reliably determine the population and subpopulation composition of lymphocytes, to detect the stage of differentiation and activation of cells, to assess the level of functional activity of lymphocytes, to determine intracellular and secreted cytokines, to conduct phagocytosis studies, to analyze the cell cycle, and to assess apoptosis and proliferation [67,68,69,70].

Decrease in the absolute number of T-lymphocytes indicates insufficiency of cellular immunity (insufficiency of the cellular-effector link of immunity), which is quite common in various infections, in nonspecific inflammatory processes, in malignant neoplasms, in the postoperative period, in infarction, etc. An increase in the number of T-lymphocytes is a prognostically favorable criterion in the dynamics of the disease and is associated with a favorable outcome. Complete termination of the disease is usually accompanied by normalisation of the T-lymphocyte count [65].

An increase in the absolute number of CD4+ T-lymphocytes indicates stimulation of the immune system to the antigen and serves as confirmation of syndrome’s hyperreactivity. It should be noted that their increase is most often a normal physiological reaction to antigen, which we observe in specific and nonspecific infectious diseases. The proliferation of CD4+ T-lymphocytes continues for 3–5 days after activation. It provides multiplication of the number of cells in clones participating in the immune response—clonal expansion of antigen-specific T cells. T cells undergo 6–8 divisions, providing an increase in their number by about 100–200 times. The initial number of T cells in a human clone can be estimated at about 2 × 10^3^ (based on an estimate of the total number of T-helper cells at 7 × 10^10^ and a possible number of clones at 3 × 10^7^); then, after proliferation, their number can exceed 106. This ensures proper efficiency of the immune response, since the formation of active T-helpers is necessary for the successful realization of practically all its branches [4,11,21]. A decrease in the number of T-helper cells indicates a hyporesponsive syndrome with a violation of the regulatory link of immunity. These changes are particularly clearly manifested against the background of HIV infection.

An increase in the number of cytotoxic T-lymphocytes is observed in almost all viral, bacterial, and protozoal infections. A relative increase in the number of CD8+ T cells is usually accompanied by a decrease in the number of T-helper cells; however, this pattern is not always observed. These changes are related to the fact that cytotoxic T-lymphocytes produce IFN-γ, which suppresses the proliferation of Th2 cells, as well as to the fact that CD8+ T-lymphocytes were previously called “T-suppressors”. The decrease in the number of cytotoxic CD8+ T-lymphocytes confirms the insufficiency of the cellular-effector link of immunity, which is especially important in the treatment of chronic viral infections (viral hepatitis, herpes, etc.) [22,23,24].

The number of B-lymphocytes in the peripheral blood is determined by the CD19 marker, which is present on all B cells in the peripheral blood but absent on plasma cells. High numbers of the absolute CD19+ B cells can be closely linked with general B-cell lymphocytes’ stimulation, which occurs during acute virus infection (EBV, COVID-19, etc.), infection with different bacteria, lymphadenopathy, and autoimmune diseases. Low levels of peripheral blood B cell can be a sign of general B-cell lymphocytopenia as a result of primary B-cell immunodeficiencies, chronic infections, and therapies with glucocorticoids or B-cell-depleting antibodies [71].

NK cells, from a diagnostic point of view, have two important CD markers—CD16 and CD16 and plasma cells. The CD markers are CD16 and CD56. Their total number in the blood is CD16+ cells—6–26%; and CD56+ cells—7–31% (0.09–0.6 × 10^9^/L). A decrease in the number of these cells is a pathognomonic sign of cellular-effector immunodeficiency associated with the severity of the course of cancer and viral infections, which is also observed when taking immunosuppressants [11,25]. An increase in the number of NK cells is associated with the activation of anti-transplantation immunity and, in some cases, is observed in bronchial asthma: it is a pathognomonic sign of cell-mediated cytotoxicity [72,73,74,75].

Currently, the so-called “regulatory” (“differentiation”) index—the ratio of CD4+ and CD8+ T-lymphocytes—has lost its clinical significance. It is considered that the value of this index less than 1.0 corresponds to immunodeficiency, and more than 2.5 corresponds to hyperactivity. However, the absolute number of T-lymphocyte subpopulations and activation markers is more informative for such conclusions [11,76].

Activation is manifested by the expression of various activation markers on cells. Thus, 2–3 h after stimulation, CD69, the earliest activation antigen, partly mobilized from intracellular depots and partly expressed de novo, appears on the surface of T-lymphocytes. Its expression continues for a little more than a day. Soon after CD69, another early marker of activation, CD25, appears on the cell surface. The next manifestations of activation are observed a day after the stimulant action, when the receptor molecule for transferrin (CD71) is expressed. In the following days (3–6 days), HLA-DRs—MHC class II molecules—referred to as late markers of T-cell activation, are expressed, and then integrins, designated as very late activation antigens (VLAs), and chemokines are secreted [26,27]. These late manifestations of cell activation are combined with the proliferative process.

The number of cells expressing receptors to IL-2 (CD25+-lymphocytes) indicates the functional state of T-lymphocytes. In normal blood, their relative number is 13–24%. In hyperreactive syndromes, the number of these cells increases, and in immunodeficiencies, it decreases. Importantly, this group is presented by T-regulators, which are responsible for peripheral immune tolerance [44,77]. The number of lymphocytes carrying two receptors—CD3 and HLA-DR—is also an indicator of immune hyperreactivity. In healthy donors, they should be no more than 12%. Currently, other markers are also being typed, and there are about 360 variants of these markers. This is especially important in oncohematology [21].

In addition to characterizing the quantitative composition of immune system cells, it is important to qualitatively characterize their functional activity. Thanks to the recent development of multicolor flow cytometry, the functional activity of cells can be assessed by the presence of specific receptors.

From the clinical point of view, the following receptors are the most important (Table 3).

To characterize the cellular component of the immune system, it is important to determine the concentration of cytokines. The determination of this immune component allows for a more accurate diagnosis and identification of the mechanism of immune disorders. The presence of proinflammatory cytokines such as TNF-α, IL-1β, and IFN-γ is of great importance, especially in the etiopathogenesis of various acute and chronic inflammatory processes of both an infectious and autoimmune nature. Their increased formation is the main cause of septic shock. In sepsis, the level of TNF-α in the blood can reach 1 ng/mL. This cytokine, acting on the hypothalamus, causes predominance of catabolic metabolic reactions over anabolic ones, and its level is elevated in neurogenic anorexia; and wound, tumor, cardiac, and infectious types of cachexia. At the postreceptor level, TNF-α interferes with the action of insulin [11].

Data on the role of proinflammatory cytokines in pathogenesis of nonspecific ulcerative colitis, multiple sclerosis, rheumatoid arthritis, insulin-dependent diabetes, etc., are accumulating [87,88]. Essential is their determination in patients with relevant congenital immunodeficiency. And if earlier tests for cytokine determination were useful only for research purposes, nowadays they become important indicators to identify the course of the disease and treatment tactics (especially when using cytokine therapy methods).

Thus, the state laboratory study of the cellular component of the immune system allows us to clarify the level of immunity disorders and subsequently conduct effective immunoactive therapy.

These indicators reflect the degree of differentiation of T- and B-lymphocytes. In the process of T- and B-lymphocyte maturation in the thymus and bone marrow, cell receptors are formed through recombination of genes in the episomal DNA chain to create a unique antigen-recognizing site. During each such recombination, a small fragment is excised from the chain to form an excision ring. These rings have been named TREC (T-Cell Receptor Excision Circle) and KREC (Kappa-Deleting Recombination Excision Circle) [39,40,41]. TRECs accompany the maturation of almost all T-lymphocytes and KRECs accompany the maturation of all B-lymphocytes and thus are markers of their number.

A decrease in TREC and/or KREC is indicative of impaired cellular and/or humoral immunity. Adults with lacking TREC and KREC deserve special attention, as studies have shown that this group has a very high risk of death during the acute period of disease. As a preliminary assessment of T- and B-lymphocyte differentiation disorders, in the absence of the necessary equipment (flow cytometer), TREC and KREC can be determined together with a complete blood count [80,87]. Russian test systems, like “IMMUNO-BIT” (for quantitative detection of TREC and KREC DNA in children up to 18 years of age) and “TK-SMA”, with an additional channel for qualitative detection of SMN1 gene deletion associated with spinal muscular atrophy (used as part of extended neonatal screening) have been developed and registered in Russia. In parallel, pilot studies on the possible application of this method in adults are being conducted in Russia and worldwide. The accumulated data allow us to state that TREC and KREC may be new biomarkers of the severity of the course and outcome of the disease, as well as monitoring the effectiveness of treatment of such pathological conditions as COVID-19, tuberculosis, malignant neoplasms, autoimmune diseases, and others [88,89,90].

### 8.2. Assessment of Indicators of Humoral Immunity

Determination of immunoglobulin levels is still an important and reliable method of assessing humoral immunity. It can be considered the main method of diagnosing all forms of immunodeficiencies associated with insufficient antibody biosynthesis, i.e., with the disorders of B-cell metabolism.

Changes in the concentration of immunoglobulins serve to confirm humoral associated forms of immunopathology. Decrease in such a concentration in the serum of patients may indicate various forms of pathology: from the inborn defects of immunoglobulin biosynthesis to transient conditions associated with the loss of protein by the organism (humoral-effector immunodeficiency). Increase in their concentrations above the normal values may indicate the presence of allergic or autoimmune processes (for example, ADCC). It is characteristic of infectious diseases at certain stages of their development (increase in IgM—in the acute period of the disease and/or exacerbation of chronic infection; gain of IgG—in the stage of resolution and/or formation of chronic infection). In addition, this method gives a criterion for the effectiveness of treatment, including substitution therapy with immunoglobulin-containing drugs.

Diagnosis of IgG subclasses is important because deficiencies of immunoglobulin subclasses may be observed when total IgG levels are normal. In some situations, immunodeficiency states are observed, manifested by increased infectious morbidity. For example, IgG2 subclass of immunoglobulin G mainly contains antibodies against polysaccharides of encapsulated bacteria (Haemophiluls influlenzae and Steptococculs pneumoniae), so deficiency associated with IgG2, as well as with IgA, leads to increased incidence of respiratory infections. Disturbances in the ratio of IgA subclasses and in the ratio of κ- and λ-chains may also be the cause of immunodeficiency states [44].

Systemic multiorgan inflammatory-fibrosing disease with hyperproduction of immunoglobulins, including autoantibodies of the IgG4 subclass (IgG4-associated immunopathy), is characteristic for early excess of immunoglobulins of this subclass in the blood, so the analysis for IgG4 gives the possibility of early laboratory diagnosis before the pronounced manifestations of organ fibrosis [91].

Levels of serum immunoglobulins (IgM, IgG1, and IgG3) reach normal values characteristic of adults just in the early postnatal period. The concentrations of IgG2, IgG4, and IgA do not reach normal adult values even during puberty. The distribution of IgG subclasses in adult serum is as follows: IgG1—60–65%; IgG2—20–25%; IgG3—10–20%; and IgG4—10–20%. Determination of the level of IgG subclasses is essential while diagnosing the hypersensitivity to bacterial infections. Deficiencies are established for almost all immunoglobulins. The most frequent are associations of IgG2, IgG4, IgA, and IgE deficiencies. The most significant deficiency is that of IgG2. It is often combined with a complete absence of IgA.

The determination of specific antibodies to various antigens provides important information on the state of humoral immunity, since the degree of the organism’s resistance to particular infection depends not on the total level of immunoglobulins but on the number of specific antibodies to its pathogen. Currently, there are a large number of test systems to recognize the level of antibodies to bacterial, viral, and fungal infections and invasions.

Determination of total IgE level is essential for differential diagnosis of atopic diseases. High levels of IgE in umbilical cord blood may be useful as an early indicator of high risk of atopic diseases.

An autoimmune process can be diagnosed in patients when certain autoantibodies are detected in the blood serum in excessive amounts [92,93]. Otherwise, the autoimmune genesis of the diseases may be questioned, which will have a significant impact on the course of further investigation and treatment. However, it should be remembered that autoantibodies may exist in low titers in sera of healthy individuals [94]. Moreover, some autoimmunopathies (e.g., Hashimoto’s thyroiditis) mainly have a pathogenesis related to T-cell mechanisms of autoaggression, and therefore their course and severity do not correlate with autoantibody levels, even with 10–12% of seronegative cases [95].

Great progress in this field is associated with the development of immunofluorescence detection of autoantibodies to various nuclear and ribosomal antigens on commercially available preparations of human HEp2 cells using fluorescein-labeled antibodies to human immunoglobulins, since in the presence of autoantibodies of different specificity, the patient’s serum gives different luminescence patterns [94]. In addition to expert evaluation, this method allows the use of artificial intelligence, which accelerates and the process and removes the subjectivity of judgement.

Antibodies to native and denatured DNA in serum are also detected by ELISA on solid-phase media. DNA, as an antigen, is sorbed on plastic, and it specifically interacts with the autoantibodies to DNA contained in the tested serum. Detection of autoantibodies to native and denatured DNA is of diagnostic value in systemic connective tissue diseases, active inflammatory processes, chronic hepatitis, infective endocarditis, and many other diseases accompanied by autoimmune processes. The presence of autoantibodies to double-strand DNA in various diseases, along with clinical manifestations, can serve as evidence of the autoimmune process.

Various immunodiagnostic methods made it possible to find specific autoantibodies to antigens of the heart, lung, kidney, liver, large and small intestine, and endocrine glands, as well as to non-organ-specific antigens, such as elastin and collagen, chromatin proteins, cytoskeleton elements, mitochondrial proteins and lipids, etc. With more than 90 autoimmune diseases of all organs and tissues having been described, the need for such diagnostic tools is growing. Rheumatology is transforming from a discipline focused on the diagnosis, treatment, and prevention of rheumatism and “rheumatism-like diseases”, as it was in the middle of the last century, into Autoimmunology, dealing with broader circle of diseases [96,97,98].

## 9. Conclusions

Thus, this review has focused on immune cells and soluble molecules involved in the pathogenesis of a wide range of diseases. But at present, we can claim that the pathogenesis of the vast majority of immune-mediated diseases is not yet known. Using clinical research methods such as hematology, flow cytometry, ELISA, etc., available to most clinical laboratories worldwide, clinical data are being collected on immune system dysfunction in a wide range of diseases. This process, unfortunately, is still very far from completing. However, the current success in dividing immune-mediated diseases into distinct clusters based on different types of inflammatory responses which are based on the involvement of different populations of T-helper cells and cytokine molecules represents significant progress. Further research in this direction seems to be very promising, as it allows for the identification of new target cells and target molecules for both improving the quality of diagnosis and targeting therapy.

## Figures and Tables

**Table 1:** Criteria for systemic inflammatory response syndrome.

Indication	Values	
Body temperature	≥38 °C	or ≤36 °C
Heart rate	≥90/min (tachycardia)	
Respiratory rate	≥20/min or blood carbon dioxide ≤ 32 mmHg	
Clinical blood analysis	Leukocytosis > 12 × 10^9^/L or young forms of granulocytes more than 10%	or leucopenia <4 × 10^9^/L

**Table 2:** Clinical manifestations of cytokine dysregulation.

Symptoms	Indicator
Fever	Maximum rise in body temperature > 38.5 °C or ≤36 °C (hypothermia)
Tachycardia	≥90/min
Respiratory failure	Dyspnea ≥ 20/min, blood carbon dioxide ≤ 32 mmHg, or saturation less than 95%
Hepatomegaly/splenomegaly	Liver and/or spleen palpated below the edge of the costal arch
Leukocytosis	>12 × 10^9^/L
Cytopenia with involvement of more than 2 cell sprouts	Hb < 90 g/L, neutrophils < 1 × 10^9^/L
Lymphocytopenia	Platelets < 100 × 10^9^/L
NK cells, CTL	Less than 1.0 × 10^9^/L
Cytokines IL-6, IL-1β, TNF, IFNγ	Elevated
Level of soluble IL-2 (CD25)	>2400 units/mL
IFNγ-induced chemokine CXCL9	Elevated
C-reactive protein	More than 5 μg/L
Serum ferritin	>500 µg/L
LDH, AST, ALT	Elevated, associated with tissue lesions
Hemostasis	Fibrinogen < 1.5 g/L, D-dimer > 243 ng/mL
Hypertriglyceridemia	Fasting triglycerides > 3.0 mmol/L or >3 standard deviations above age-appropriate levels
Procalcitonin	Above 2.0 ng/mL
Hemophagocytosis	In bone marrow, spleen, or lymph node biopsies

**Table 3:** Important immunological receptors associated with clinical data.

Immunological Receptors	Functions
CD5	CD5 is an adhesion molecule that regulates cell activation. It is detected on T-lymphocytes, thymocytes, and B1-clone of B cells.
CD11b	CD11b belongs to the most important integrins for cell migration that determine the activity of phagocytosis, cell cytotoxicity, chemotaxis, and cellular activation of T effectors, NK cells, macrophages, and granulocytes [5,67].
CD16	CD16 is a receptor of the Fc-fragment of IgG, it mediates phagocytosis and antibody-dependent cellular cytotoxicity, its activation increases the cytotoxicity of NK cells, and it stimulates the secretion of IFN and TNF-α [5,77,78,79].
CD23	CD23 is expressed on activated B cells, macrophages, thymic epithelial cells, eosinophils, and platelets. It is also an indicator of B-cell activity [5,80].
CD25	CD25 is the α-chain of IL-2 receptor. It is expressed on different types of peripheral blood cells: CD4+-, CD8+-, NK-lymphocytes, NKT cells, B-lymphocytes, and monocytes. Marker of early activation of T-lymphocytes. An increase in their number, as well as in the total population of CD25-positive lymphocytes, may indicate an inflammatory process of any nature (infectious and autoimmune) [5,81].
CD27	CD27 is an additional marker of B2-lymphocytes and indicates the transition of B-lymphocytes from naive cells to memory cells.
CD28	CD28 is expressed on most activated T-lymphocytes, naive T cells and memory T cells. It is required as a costimulatory factor for induction of immune response (cell proliferation and activation) [5,82,83].
CD38	CD38, cyclic ADP-ribosylhydrolase, located on the surface of lymphocytes, provides adhesion and signal transduction; it is also a marker of cell activation (metabolic marker). It is decreased in HIV infection, leukemia, myeloma, co-lead tumors, and type II diabetes mellitus [5,82].
CD50	CD50 is an intercellular adhesion molecule (ICAM-3), in addition to being a potent signaling molecule. Present on all leukocytes, endothelial, and dendritic cells. Provides costimulatory signals for T cells and regulates cell adhesion by interacting with integrins. It has been shown to decrease the number of CD50+ cells in tumor diseases [5,81].
CD57	CD57 is expressed on subpopulations of 15–20% of peripheral blood mononuclear cells, and in 60% of NK- and T cells. Elevated values are found in cancer patients, post-transplant patients, patients with HIV, and patients with rheumatoid arthritis and Felty’s syndrome. The decrease is pathognomonic for chronic Lyme disease [4,84].
CD62L	CD62L is a member of the family of cell adhesion molecules (L-selectin) located on the cell surface of leukocytes (T and NK cells, monocytes, and granulocytes); it provides translocation of leukocytes from the blood to the lymphoid tissue, where they interact with antigen [4,85].
CD64	CD64 is a mediator of antibody-dependent cellular cytotoxicity (functional marker).
CD158a	CD158a is an important functional marker of NK activity.
HLA-DR	HLA-DR is expressed by various cells of peripheral blood. It is detected on all B-lymphocytes and monocytes, and on activated T-lymphocytes (marker of late activation), but it also plays an important part in monocytes functions. The level of HLA-DR-receptor expression on monocytes less than 50% is an unfavorable pathognomonic sign of severe bacterial infection (sepsis, peritonitis) [4,86].

## Data Availability

Not applicable.
